# Intraoperative Neuromonitoring for Cerebral Arteriovenous Malformation Embolization: A Propensity-Score Matched Retrospective Database Study

**DOI:** 10.7759/cureus.12946

**Published:** 2021-01-27

**Authors:** Austin Y Feng, Eric S Sussman, Michael C Jin, Sandy Wong, Jaime Lopez, Benjamin Pulli, Jeremy J Heit, Nicholas Telischak

**Affiliations:** 1 Neurosurgery, Stanford University School of Medicine, Stanford, USA; 2 Neurology, Stanford University School of Medicine, Stanford, USA; 3 Radiology, Stanford University School of Medicine, Stanford, USA

**Keywords:** intraoperative neuromonitoring, arteriovenous malformations, marketscan

## Abstract

Introduction

The treatment of cerebral arteriovenous malformations (AVMs) may result in neurologic morbidity, particularly when an AVM is located in or adjacent to eloquent brain regions. Intraoperative neurophysiologic monitoring (IONM) may be utilized to reduce the risk of iatrogenic injury during endovascular AVM embolization; however, IONM for endovascular AVM embolization is not ubiquitously the standard of care.

Methods

Admissions for AVM embolization were assessed from the IBM MarketScan® Commercial and Medicare Supplemental databases (IBM Watson Health, Somers, NY). Inclusion criterion for patients was continuous enrollment six months before and after the index encounter. The use of IONM and presence of intracranial hemorrhage (ICH) were noted. Propensity-score matched cohorts with and without IONM were generated to minimize bias between treatment groups (adjusting for age, sex, and comorbidities).

Results

From 2007 to 2016, there were 16,279 patients diagnosed with cerebral AVM in the MarketScan database. Embolized patients were stratified into IONM and non-IONM cohorts; there were 357 patients in the IONM cohort and 1775 patients in the non-IONM cohort. Provider types were significantly different between cohorts (p<0.005). Unruptured AVMs were significantly more likely to be embolized with adjunctive IONM (17.7%) compared to ruptured AVMs (7.9%) (p<0.005). After balancing for baseline comorbidities, there were 266 patients in the IONM cohort, and 1347 patients in the non-IONM cohort. Among unruptured AVM patients, IONM was linked to a significantly shorter length of stay (2.72 versus 4.92 days; p<0.005), significantly lower rates of complications within 30 days of discharge (0.00% versus 1.88%; p=0.038), and significantly lower total payment ($40,179 versus $50,844; p<0.0001).

Conclusion

Endovascular embolization for unruptured AVMs performed with adjunctive IONM was associated with shorter length of stay, lower complication rates, and hospitalization costs.

## Introduction

Intracranial arteriovenous malformations (AVMs) are congenital vascular lesions associated with a significant risk of lifelong morbidity primarily due to the risk of hemorrhage and seizures. Treatment of these lesions carries a risk of iatrogenic injury to eloquent or deep brain regions. Meta-analyses of large AVM case series indicate that severe adverse events may occur in up to 7.4% of patients undergoing microsurgery for AVM resection [1]. Given the significant potential morbidity of AVM treatment, the management of incidentally discovered or minimally symptomatic AVMs requires balancing possible operative risks with the potential benefits of curative treatment. Given these complex and idiosyncratic considerations, there is no clear consensus regarding treatment.

Intraoperative neurophysiologic monitoring (IONM) utilizes electrophysiologic techniques of spontaneous or evoked neural activity to assess the functional status of a patient’s nervous system during operative procedures. Such monitoring can alert the operative team of impending insults to neural integrity and allow real-time adjustment to mitigate risk. Improvements in IONM techniques have increasingly led to their adoption in a variety of neurosurgical procedures [2-4]. Especially for AVMs, IONM offers many advantages given their high risk. However, its use in AVM treatments is not widespread or well characterized. Compared to the literature for other neurosurgical procedures, there is far less evidence of IONM efficacy in AVM treatment, particularly embolization. The literature is sparse at best, and is primarily composed of case series with anecdotal accounts of IONM use [5-10]. Clinically relevant questions regarding length of stay, readmission rates, discharge status, and costs remain unanswered. A large-scale comprehensive analysis could address these unknowns and could improve our understanding of IONM utility during AVM surgery. Here, we queried a national database to investigate the utilization and effectiveness of IONM as an adjunct during endovascular AVM embolization.

## Materials and methods

A retrospective analysis of IONM for AVM embolization on a population level was performed to elucidate the impact of IONM on outcomes and complications. National patient data were acquired using the MarketScan® Commercial Claims and Encounters and Medicare Supplemental databases (IBM Watson Health, Somers, NY). As we previously described, the MarketScan database tracks information of millions of Americans annually from over 100 payers in the private sector as well as Medicare.

International Classification of Diseases, 9th/10th Revision (ICD-9/ICD-10) and Current Procedural Terminology (CPT) codes were used to identify diagnoses and procedures in patient records. Patients with intracranial AVM diagnoses were identified with the ICD-9/ICD-10 codes 747.81/Q282. The embolization of AVMs was identified with the CPT and ICD-9 codes 61624 and 39.72, respectively. Concurrent IONM was noted by the G-code G0453 and CPT codes 95940, 95941, and 95920 associated with the embolization procedure. Intracranial hemorrhage (ICH) was noted by the ICD-9/ICD-10 codes 431 and I61.9. Inclusion criteria for patient records were intracranial AVM diagnosis with embolization. The first record of both diagnosis and procedure was defined as the index encounter. Patients with insufficient or incomplete continuous enrollment of at least six months before and after this index encounter were excluded.

Cohorts with and without IONM were compared using a bivariate analysis. Propensity score matching was used to balance baseline comorbidities between patients in order to minimize biases that may increase or decrease the likelihood of receiving IONM. Using a greedy matching algorithm, matching was performed without replacement. The caliper was chosen as 20% of the standard deviation of the propensity score logit. Matching covariates include demographics (age and sex) and other common comorbidities previously reported (e.g., diabetes mellitus, hypertension, congestive obstructive pulmonary disease, tobacco use, obesity, heart failure, rheumatic disorders, and drug abuse) [11,12]. Patients with IONM were matched to patients without IONM at a ratio of 1:5. After matching, nearly all covariate differences were insignificant and all standardized mean differences were less than 0.1 (Table 1).

**Table 1:** Propensity-Score Matched Cohorts IONM, intraoperative neurophysiologic monitoring; COPD, chronic obstructive pulmonary disease

Covariates	IONM		non-IONM		
	n	%	n	%	p
Age	44.9	-	46.3	-	0.303
Sex (female)	123	56.4	832	59.8	0.349
Diabetes	7	3.2	55	4.0	0.570
Hypertension	10	4.6	70	5.0	0.772
COPD	7	3.2	27	1.9	0.312
Tobacco	22	10.1	141	10.1	0.984
Obesity	7	3.2	40	2.9	0.793
Heart failure	0	0	5	0.4	0.025
Rheumatic disease	0	0	12	0.9	<0.001
Drug abuse	0	0	0	0	1

Assessed outcomes from matched cohorts included discharge status, all-cause readmission rate within 30 days, length of stay, and postoperative adverse events within 30 days. The distribution of primary interventionalist analysis was performed on pre-matched cohorts. Complications were defined by ICD-9/ICD-10 diagnoses of any postoperative neurological complication, wound complication or infection, delirium, chronic pain, hydrocephalus, stroke, altered mental status, disorientation, seizure, headache, septicemia, cognitive deficit, and/or dysrhythmia. To ensure complications were postoperative, patients could not have the same ICD-9/ICD-10 diagnoses within the prior six months. Categorical variables were analyzed using the Fisher exact test. Continuous variables were analyzed using the Student’s t-test. Statistical significance was established at p-value less than 0.05. Analyses were performed using R software, version 3.6.3 (R Foundation for Statistical Computing, Vienna, Austria).

## Results

Patients

From 2007 to 2016, there were a total of 16,279 patients with intracranial AVM diagnoses in the MarketScan database, of which 3043 had ruptured AVMs and 13,236 had unruptured AVMs. About 13.1% of intracranial AVM patients (N=2132) underwent endovascular embolization, and they were stratified according to AVM rupture status: 1775 patients in the unruptured AVM cohort and 357 patients in the ruptured AVM cohort. Unruptured AVMs were significantly more likely to be embolized with adjunctive IONM (17.7%) compared to ruptured AVMs (7.9%) (p<0.001) (Table 2).

**Table 2:** Provider Type and AVM Rupture Status AVM, arteriovenous malformation; IONM, intraoperative neurophysiologic monitoring; ICH, intracranial hemorrhage; N/A, not applicable

	IONM		non-IONM			ICH		non-ICH		
	n	%	n	%	p	n	%	n	%	p
Provider					0.019					0.772
Radiology	9	2.5	276	15.6		26	11.7	259	13.6	
Neurology	148	41.5	123	6.9		32	14.4	239	12.5	
Neurosurgery	34	9.5	218	12.3		30	13.5	222	11.6	
MD (other)	151	42.3	1076	60.6		124	55.9	1103	57.7	
N/A	15	4.2	82	4.6		10	4.5	87	4.6	
Rupture status					<0.001					
ICH	19		203							
non-ICH	338		1572							

In 37.9% of cases, the listed interventionalist was either a radiologist (13.4%), neurologist (12.7%), or a neurosurgeon (11.8%), with the remaining 62.1% noted as unlisted or other provider type (Table 2). In the pre-matched cohort, there was a statistically significant difference in provider type between IONM cohorts (p=0.019); neurologists were the most common interventionalist in the IONM cohort, and radiologists were the most common provider type in the non-IONM cohort. The frequency of neurosurgeons was not different between both groups (p=0.141). Lastly, provider types were not significantly different between ruptured and unruptured AVM (p=0.772).

After selecting balanced cohorts for baseline comorbidities, there were 220 patients who received IONM and 1393 who did not receive IONM. Among patients with ruptured AVMs, 7.1% (N=19) received IONM and 88.3% (N=247) did not receive IONM during treatment. Among unruptured AVM patients, 14.9% (N=201) received IONM and 85.1% (N=1146) did not receive IONM during treatment. There were 266 patients who had ruptured AVMs, with 7.1% (N=19) and 88.3% (N=247) who did and did not receive IONM, respectively. There were 1347 patients with unruptured AVMs, with 14.9% (N=201) and 85.1% (N=1146) who did and did not receive IONM, respectively.

Discharge status

In the overall cohort, there was no significant difference in rates of home discharge and death between the IONM and non-IONM subgroups (89.6% versus 83.8%, and 0.5% versus 1.5%, respectively; p=0.265). There was a significantly lower rate of home discharges in all patients undergoing embolization of ruptured AVMs compared to patients undergoing embolization of unruptured AVMs (63.8% versus 88.4%; p<0.001). Rates of death were significantly greater in all patients with ruptured compared to unruptured AVMs (5.9% versus 0.6%; p<0.001). However, among ruptured AVM patients, IONM status was not statistically associated with discharge status (p=0.927). Similarly, no statistically significant relationship was found between IONM and discharge statuses for the unruptured cohort (p=0.803) (Table 3).

**Table 3:** Details of Discharge Status, Length of Stay, and 30-Day Readmission and Postoperative Complication Rates IONM, intraoperative neurophysiologic monitoring; ICH, intracranial hemorrhage; N/A, not applicable

	Overall						ICH							Non-ICH							
	IONM			non-IONM			IONM			non-IONM				IONM			non-IONM				
	n	%		n	%	p	n		%	n	%		p	n		%	n		%		p
Discharge						0.265							0.927								0.803
Home	190	89.6		1091	83.8		14		77.7	136	62.7			176		90.7	955		87.9		
Skilled nursing	2	0.9		20	1.5		0		0	8	3.7			2		1	12		1.1		
Death	1	0.5		20	1.5		1		5.6	12	5.5			0		0	8		0.7		
Hospice	0	0		5	0.4		0		0	2	0.9			0		0	3		0.3		
Inpatient rehab facility	5	2.4		91	7		2		11.1	44	20.3			3		1.6	47		4.3		
Long-term care hospital	0	0		13	1		0		0	5	2.3			0		0	8		0.7		
N/A	14	6.6		63	4.8		1		5.6	10	4.6			13		6.7	53		4.9		
Length of stay (days)	2.9			6.6		<0.001	5.3			14.3			<0.001	2.7			4.9				<0.001
30-day readmission rate	7.7			9.7		0.46	15.8			4			0.061	7			10.9				0.133
30-day postoperative complication (any)	0	0		23	1.7	0.04	0		0	2	0.8		1	0		0	22		1.9		0.038

Length of stay/30-day readmission rate

IONM was statistically associated with shorter length of hospital stay (6.6 versus 2.9 days; p<0.001). Furthermore, length of stay was significantly shorter in the IONM cohort of both ruptured (5.32 versus 14.3 days; p<0.001) and unruptured (2.7 versus 4.9 days; p<0.001) AVM subgroups. Between ruptured and unruptured AVM groups, different non-significant trends were associated with IONM use. The IONM cohort of ruptured AVMs had a higher 30-day readmission rate (15.8% versus 4.0%; p=0.061), while the IONM cohort of unruptured AVM had a lower rate (7.0% versus 10.9%; p=0.133) (Table 3).

Related costs

Overall, the mean total payment per patient index encounter was significantly lower in the IONM cohort compared to the non-IONM cohort ($43,764 versus $54,667; p<0.001). On a more granular level, the hospital payment per patient index encounter was also significantly lower for the IONM cohort ($36,721 versus $46,467; p<0.001), and there was a trend towards lower physician professional payments, which did not reach statistical significance ($3678 versus $4111 in the non-IONM group; p=0.129).

Among only patients with unruptured AVMs, IONM was also associated with significantly lower mean total payment ($40,179 versus $50,844 in the non-IONM group; p<0.001), including significantly lower mean hospital payments ($33,336 versus $42,963; p<0.001), and a non-significant trend towards lower physician professional payments ($3558 versus $4044; p=0.10) in the IONM subgroup. In the sub-analysis of the ruptured AVM subgroup, overall costs were substantially higher than in the unruptured AVM subgroup; however, there were no significant differences in mean total ($82,639 versus $75,857; p=0.659), hospital ($73,428 versus $65,897; p=0.604), or physician professional ($4982 versus $4482; p=0.615) payments between IONM and non-IONM subgroups, respectively (Table 4).

**Table 4:** Total Costs IONM, intraoperative neurophysiologic monitoring; ICH, intracranial hemorrhage

	Overall					ICH					non-ICH				
	IONM		non-IONM			IONM		non-IONM			IONM		non-IONM		
	Mean	SD	Mean	SD	p	Mean	SD	Mean	SD	p	Mean	SD	Mean	SD	p
Payment ($)															
Total	43,764	59701	54,667	82150	<0.001	82,639	116651	75,857	114585	0.659	40,179	50039	50,844	74201	<0.001
Hospital	36,721	54359	46,467	74462	<0.001	73,428	110417	65,897	105002	0.604	33,336	44537	42,963	66923	<0.001
Physician	3678	6667	4111	9342	0.129	4982	7337	4482	9133	0.615	3558	6594	4044	9379	0.105

Overall postoperative complication (30-day)

The 30-day postoperative complication rates were lower in IONM cohorts for unruptured AVMs. For the overall analysis, IONM was associated with significantly lower overall complication rates (0.0% versus 1.7%; p=0.039). Postoperative complication rates were not significantly lower for the IONM cohort for ruptured AVMs (p=1.00), but were in patients with unruptured AVMs (p=0.038). Hydrocephalus and stroke were the most common complications in each cohort, respectively (Table 3).

## Discussion

In this study, we performed the first retrospective analysis of patients with intracranial AVMs who received embolization with and without IONM from nationally sourced real-world data. The general utility of IONM has been explored in other neurosurgical procedures, ranging from benefits observed in spinal tumors and thoracolumbar fixation, to no benefit or even detriment seen in anterior cervical discectomy and fusions, as well as in pediatric procedures [2,6,13,14]. However, the value of IONM has only been minimally explored and reported for endovascular procedures, much less for intracranial AVM embolization [6,15]. Particularly for AVMs, their comparatively low incidence combined with the complexity of both their pathology and surgical management likely contributes to the current lack of guidelines regarding IONM usage for their treatment [16].

Despite the risks of hemorrhage in untreated AVMs, embolization can carry substantial risks. In a review of latter 20th-century AVM case series, Frizzel and Fisher reported rates of permanent morbidity between 8% and 9% from embolization [17]. In comparison, a meta-analysis by van Beijnum et al. of AVM case series from 2000 to 2011 found serious complications leading to permanent neurological damage or death in 6.6% of cases [1]. Although these findings demonstrate that there have been advances in embolization safety, there is still room for improvement. The routine use of IONM may be such an avenue of further mitigating complications.

Zhou et al. performed a retrospective cohort study of postoperative neurologic complications to analyze the effectiveness of IONM during AVM surgery. Sensitivity and specificity for the prediction of short-term neurologic dysfunction were 86.7% and 100%, respectively, which suggests that IONM was a highly reliable tool [5]. In a study by Paulsen et al., 17 awake patients with rolandic AVMs underwent sodium amobarbital provocation testing (PT) with clinical examination and somatosensory-evoked potential (SEP) during embolization. With positive SEPs with amobarbital PTs excluding two patients, the remaining patients were embolized. Though four patients had temporary deficits, none developed any permanent neurological deficits [7]. Rauch et al. reported on the incidence of complications following supratentorial AVM embolization in 30 patients who underwent amobarbital PT with both clinical and EEG monitoring; 82 embolizations performed following negative PTs without IONM changes were linked to no neurological complications. They also demonstrated the importance of IONM, whereby clinical examination detected only half as many positive amobarbital PT as EEG [18]. Sala et al. employed SEP and motor-evoked potentials (MEPs) during lidocaine PT on 11 patients with eloquent sensory-motor region AVMs receiving embolization under general anesthesia. Only feeding arteries with negative PTs with no MEP or SEP changes were embolized and no permanent neurological events were observed [6]. Despite heterogeneity in anesthesia as well as IONM methodology and likely differences in surgical skill across procedures, these anecdotal accounts parallel our population-level results, whereby embolization with IONM was associated with a significantly lower complication rate within 30 days of discharge. Fewer complications likely contributed to the significantly shorter length of stay. Though not significantly different, the trends towards a higher rate of home discharge and lower 30-day readmission rate may be explained by lower incidence of complications.

Routine IONM use was also supported by the apparent cost-efficiency of its use. Between balanced cohorts, patients who received embolization with IONM spent almost $10,000 less in hospital fees compared those without IONM. This was likely driven by the significantly shorter length of stay associated with IONM. While we are unable to perform more precise cost and/or cost-benefit analysis of IONM with our available data, these findings are the first cost assessments of IONM for a neuroendovascular procedure to our knowledge.

Although IONM can mitigate risk in complex procedures, it is context dependent. A notable finding was that the rupture status of the AVM was linked to concurrent use of IONM during embolization, whereby IONM use was more common in unruptured AVMs. One possible explanation is the time-sensitive nature of hemorrhaging AVMs [19]. Time must be spent to not only setup IONM, but also acquire baselines for comparison. In urgent situations, delaying active treatment for the patient may pose a greater threat than that mitigated by IONM. Finally, the relative experience of the neurosurgeon or interventional radiologist may play a role, given that IONM can be used as a training tool [20].

Limitations

Although the use of the MarketScan database has numerous advantages, there are a few limitations. Information extracted from a billing and claims database lacks the granularity of electronic health records. IONM can involve a wide permutation of specific neurophysiologic modalities (e.g., MEP, SEP, EEG, etc.), but the CPT codes categorize IONM as a single entity. Both ICH and intracranial AVM characteristics also vary greatly depending on the specific lesion topography, but their description is restricted by the ICD-9/ICD-10 codes. Furthermore, centers with larger AVM patient volumes that use IONM may have a larger influence on observed data trends, but the type of institution (i.e., academic versus community) is not coded. Altogether, it is difficult to ascertain the possible confounding effects of these notable details. Analyses also depend on the accurate documentation of diagnoses, procedures, and complications. Though errors are inevitable given the wide range of patients and payers, they are likely minimized given the incentive for proper compensation. Lastly, both cohorts may have traits that are unrepresentative of the general populace. For instance, by nature of inclusion in the database, patients must have health insurance, private or Medicare.

## Conclusions

In the first study of its kind, patient cohorts from a nationally represented sample underwent propensity score matching and were evaluated for the effects of IONM on AVM embolization outcomes and complications. We found that endovascular embolization for unruptured AVMs performed with adjunctive IONM was associated with shorter length of stay and lower complication rates. We also found cost savings of nearly $10,000 per patient associated with the use of IONM. Despite its limitations, we believe our study contributes significantly to the discussion of IONM utility and suggests possible benefits from IONM use. However, additional prospective evaluation of IONM in AVM embolization is warranted.
